# K_v_1.1 channels in cardiorespiratory regulation and sudden unexpected death in epilepsy: insights from mouse models

**DOI:** 10.1093/braincomms/fcaf116

**Published:** 2025-03-20

**Authors:** Amanda Hu, Khaing Phyu Aung, Christopher A Reid, Ming S Soh

**Affiliations:** The Florey Institute of Neuroscience and Mental Health, University of Melbourne, Parkville, VIC 3052, Australia; The Florey Institute of Neuroscience and Mental Health, University of Melbourne, Parkville, VIC 3052, Australia; The Florey Institute of Neuroscience and Mental Health, University of Melbourne, Parkville, VIC 3052, Australia; The Florey Institute of Neuroscience and Mental Health, University of Melbourne, Parkville, VIC 3052, Australia

## Abstract

This scientific commentary refers to ‘Seizures and premature death in mice with targeted Kv1.1 deficiency in corticolimbic circuits’, by Paulhus and Glasscock (https://doi.org/10.1093/braincomms/fcae444).

**This scientific commentary refers to ‘Seizures and premature death in mice with targeted Kv1.1 deficiency in corticolimbic circuits’, by Paulhus and Glasscock (https://doi.org/10.1093/braincomms/fcae444).**

Patients with epilepsy are at an increased risk of premature mortality compared to healthy individuals, with sudden unexpected death in epilepsy (SUDEP) being the most common cause.^1^ Frequent generalized tonic–clonic seizure is one of the biggest risk factor, although the incidence of SUDEP can vary vastly depending on the population of interest.^1^ Changes in seizure-mediated neuro-cardio-respiratory function are thought to underlie SUDEP, although the exact sequence of events is unclear.^1^ Efforts to investigate SUDEP in human patients are challenging given its relative rarity and the unpredictability of occurrence.

MORTality in Epilepsy Monitoring Unit Study (MORTEMUS), a systematic retrospective study of near-SUDEP and SUDEP cases from 147 epilepsy monitoring units worldwide, provided a crucial insight into the mechanisms of SUDEP.^2^ Of the 16 SUDEP cases reviewed, nine of the patients with severe epilepsies were under video-electroencephalography (VEEG) and ECG monitoring at the time of death, which allows clinicians to capture seizure events and the concomitant respiratory and cardiac components. Out of the nine patients, only six had complete uninterrupted postictal recording until the time of death. In these six patients, VEEG–ECG recordings demonstrated changes in cardiorespiratory function following a seizure prior to death. In particular, it was noted that generalized tonic–clonic seizure-mediated terminal apnoea occurred before terminal asystole, strongly implicating respiratory depression as the main cause of SUDEP.^2^

The MORTEMUS conclusion is largely supported by findings in the recent study by Paulhus and Glasscock^3^ in *Brain Communications,* using a novel conditional knockout (KO) SUDEP mouse model with *Kcna1* specifically deleted in the neocortex and limbic excitatory neurons (corticolimbic *Kcna1*-KO). *Kcna1* encodes a voltage-gated potassium channel α-subunit, K_v_1.1. The channel is located both in the heart and brain, where it regulates cardiac rhythm and neuronal excitability, respectively.^4^ Variants in the human *KCNA1* have been associated with severe epilepsies.^4^ Glasscock’s team previously employed two other *Kcna1*-based mouse models to understand the mechanisms underlying epilepsy and SUDEP. One of them was a global *Kcna1*-KO model, which involved the deletion of the *Kcna1* gene in all tissues throughout the body, including both the brain and peripheral organs.^5^ The other model was a neuron-specific *Kcna1*-KO mouse in which K_v_1.1 was absent in all neurons in the central nervous system, except the cerebellum.^6^ All three *Kcna1*-based mouse models experienced generalized tonic–clonic seizures and premature death at varying rates and severity.^3,5-7^ Twenty-four-hour VEEG-ECG recordings demonstrated that the corticolimbic *Kcna1*-KO mice experienced seizure-induced cardiac arrhythmias, with heart block and bradycardia occurring in half of the seizures.^3^ Cardiac abnormalities following seizures were less common, occurring in not more than 1 out of 10 seizures. Seizure-mediated respiratory impairment was also noted during the 6-h VEEG-ECG-plethysmography recordings and was observed more frequently than cardiac dysfunction.^3^ In more than half of the captured seizures, respiratory abnormalities preceded cardiac dysfunction. This pattern was also observed during the one terminal seizure that was captured, whereby bradycardia occurred shortly following respiratory dysfunction, and asystole was noted 8 min after breathing has stopped. This observation was in line with the MORTEMUS study^2^ and consistent with a primary role of seizure-induced respiratory dysfunction—in this case, mediated through K_v_1.1-deficient corticolimbic excitatory network—in the pathophysiology of SUDEP.

Nonetheless, it is important to acknowledge that the cardiopulmonary systems are closely intertwined and the two are dependent on each other. A hypoxic environment, potentially due to seizure-induced neuro-cardiorespiratory changes, can trigger a cardiac incidence known as pulseless electrical activity (PEA), which has a high risk of mortality.^8^ PEA can also be caused by trauma and electrolyte imbalance, which can occur during a seizure.^8,9^ During PEA, the heart can maintain an organized electrical activity on the ECG, but this may not be sufficient for a full ventricular contraction, resulting in a lack of detectable pulse.^8,10^ The weakened contraction can in turn cause the respiratory system to fail, leading to a multisystem breakdown. The temporal sequence of events in the one captured spontaneous death may provide some additional insight into the cardiorespiratory changes that occur in SUDEP. The authors noted that, following the terminal seizure, heart function persisted at a bradycardic rate without recovering to baseline rhythm until it subsequently stopped, long after respiration has ceased.^3^ It is essential to highlight that PEA can sometimes be mistaken as bradycardia.^8,10^ PEA is characterized by distinct changes in ECG morphology, including the slowing of the QRS interval,^10^ something that was not analysed in this study. Therefore, in this and in other studies, the existence of an organized ECG activity at the end of a terminal seizure is not necessarily an indication of a working heart. Future studies need to not only measure ECG activity but also monitor the pulse to ensure that PEA is not a feature of the cascade leading to death in SUDEP.

Another feature of the study is the concurrent VEEG-ECG-plethysmography recording, which allows in-depth analysis of the brain, heart and lung function during a seizure. The authors methodically categorized the cardiorespiratory abnormalities that occurred during non-terminal spontaneous seizures.^3^ They concluded that respiratory events were observed more frequently compared to cardiac dysfunction.^3^ The study also noted that most seizures were of milder nature. However, it is unclear if all seizures resulted in cardiorespiratory changes, or if there is an overlap of phenotypes within a single seizure. For instance, does a single mild seizure result in sinus pauses, bradycardia and ataxic breathing? What is the percentage of severe seizure that causes both tachypnoea and tachycardia? Do longer seizures worsen cardiorespiratory dysfunction? Categorizing the seizures using an established scoring system such as the Racine scale and correlating seizure duration and severity to the respective cardiorespiratory changes could provide a clue on the interaction between the different systems. The frequency and severity relationship between cardiorespiratory abnormalities and seizures were explored in Glasscock’s previous work using global *Kcna1*-KO mice^5^ but not in the present study. Long-term VEEG-ECG-plethysmography monitoring over a course of few days should also be considered to pick-up any day-to-day variations. Any significant changes from the recording could perhaps be used as a physiological biomarker to assess risk.

While neuro-cardiorespiratory dysfunction has been thought to underlie SUDEP, the specific neurocircuits that are involved remains unclear. Compared to corticolimbic *Kcna1*-KO mice, global and neuron-specific *Kcna1*-KO mouse models both similarly exhibited frequent early-onset generalized tonic–clonic seizures, seizure-mediated cardiorespiratory abnormalities and a high incidence of premature death.^3,5,6^ This supports the critical role of centrally located neuronal K_v_1.1 channels in causing epilepsy and cardiorespiratory dysregulation, which can lead to SUDEP. The authors nevertheless pointed out that there are phenotypic differences between the three models.^3,5,6^ Firstly, global *Kcna1*-KO mice had the most severe seizure phenotypes and the highest mortality rates, followed by neuron-specific and then corticolimbic *Kcna1*-KO mice. Secondly, global *Kcna1*-KO mice exhibited the most pronounced ictal and interictal cardiorespiratory dysfunction, while corticolimbic *Kcna1*-KO mice showed dysfunction primarily during seizures. Thirdly, increased heart rate variability and lack of apnoea during interictal periods was observed in global and neuron-specific *Kcna1*-KO mice, but not in the corticolimbic KO counterpart. This suggests that, while *Kcna1* in the forebrain is involved, the varying contributions of K_v_1.1 channels in different brain regions and cell types to epilepsy, cardiorespiratory regulation and SUDEP should not be ignored.

The corticolimbic *Kcna1*-KO mice, which targets deletion of K_v_1.1 potassium channel primarily in the forebrain regions,^3^ is a valuable tool to understand epilepsy and SUDEP pathophysiology. However, like all animal models, there are limitations. SUDEP can be caused by various genetic factors—including those that encode ion channels other than K_v_1.1—and non-genetic factors, such as seizure frequency and age of onset.^1,11^ Therefore, the corticolimbic *Kcna1*-KO mouse model is unlikely to capture all the underlying mechanisms of SUDEP. Furthermore, besides the corticolimbic circuits, other brain regions and neuronal networks may also play significant roles in SUDEP.^5,6^ While seizure-induced respiratory dysfunction leading to cardiac arrest is a driving factor in many sudden death cases,^3^ SUDEP can also involve other mechanisms, such as cardiac arrhythmias.^1,11-14^ Importantly, human SUDEP cases exhibit considerable variability in terms of seizure types, frequency and associated comorbidities.^1,15^ The current model may not encompass this full spectrum of variability, limiting its generalizability to all SUDEP cases.

In summary, Paulhus and Glasscock’s study supports respiratory dysfunction as a primary factor in SUDEP, although cardiac contributions should not be disregarded. Similarly, the findings suggest that while corticolimbic K_v_1.1 channels play a role in SUDEP through cardiorespiratory dysfunction, contributions from K_v_1.1 in other parts of the body should not be overlooked. While the corticolimbic *Kcna1*-KO model is a valuable model for studying specific aspects of SUDEP, it may not capture all the diverse mechanisms and factors involved in all SUDEP cases. Further research using various models and approaches is necessary to fully understand and address the causes of SUDEP.

## Data Availability

No data was generated for this commentary.
